# Quality control in the year 2000

**DOI:** 10.1155/S1463924692000154

**Published:** 1992

**Authors:** Bernd Schade

**Affiliations:** Miles, Inc. Consumer Healthcare Division Elkhart Indiana USA

## Abstract

‘Just-in-time’ production is a prerequisite for a company to meet the challenges of competition. Manufacturing cycles have been so successfully optimized that release time now has become a significant factor. A vision for a major quality-control (QC) contribution to profitability in this decade seems to be the just-in-time release. Benefits will go beyond cost savings for lower inventory. The earlier detection of problems will reduce rejections and scrap. In addition, problem analysis and problem-solving will be easier.

To achieve just-in-time release, advanced automated systems like robots will become the workhorses in QC for high volume pharmaceutical production. The requirements for these systems are extremely high in terms of quality, reliability and ruggedness. Crucial for the success might be advances in use of microelectronics for error checks, system recording, trouble shooting, etc. as well as creative new approaches (for example the use of redundant assay systems).


Journal of Automatic Chemistry, Vol. 14, No. 2 (March–April 1992), pp. 65–67

### Quality control in the year 2000

**Bernd Schade**
*Miles, Inc., Consumer Healthcare Division, Elkhart, Indiana, USA*

*'Just-in-time' production is a prerequisite for a company to meet the challenges of competition. Manufacturing cycles have been so successfully optimized that release time now has become a significant factor. A vision for a major quality-control (QC) contribution to profitability in this decade seems to be the just-in-time release. Benefits will go beyond cost savings for lower inventory. The earlier detection of problems will reduce rejections and scrap. In addition, problem analysis and problem-solving will be easier.*

*To achieve just-in-time release, advanced automated systems like robots will become the workhorses in QC for high volume pharmaceutical production. The requirements for these systems are extremely high in terms of quality, reliability and ruggedness. Crucial for the success might be advances in use of microelectronics for error checks, system recording, trouble shooting, etc. as well as creative new approaches (for example the use of redundant assay systems).*

### Introduction

It is probable that in the future there will be some quality-control areas that will require much less testing than is needed today. The reason is, of course, that computer intelligence is so inexpensive. Many production machines have a lot of computer intelligence built in to control the machines and reduce the margin of error. For example, any modern tableting machine is able to get feedback from an automated hardness and weight-testing instrument. The feedback information from this instrument will bring the tableting machine on target again if it was drifting off from the target value. This approach means that these parameters do not have to be tested again after the batch is manufactured.

Of course, this situation is not true for all areas. There will be areas where there will be more testing to do, because of increasing regulatory requests. However, costs per test will be cheaper and therefore an increased level can be accomplished.

There is no doubt that automation will gain new breakthroughs in the 1990s and robotic systems will have a big share in this development. Robots will probably become *the* workhorses in the quality-control environment.

### The QC environment at Miles

When the benefits of robots were evaluated in the Consumer Health Care Division at Miles, it was decided to start in the Quality Control unit rather than in the research area: there is so much routine work to do in quality control that the payback here was likely to be the highest. Miles is best known for the Alka Seltzer and Alka Seltzer Plus products; there are about seven different products manufactured in this family. In several respects, they are different from other tablets. The tablet size is big, about 1" in diameter. They are moisture sensitive so they are packaged in aluminium, and they are packaged immediately after compression. Over 150 batches per week are made: each batch is somewhat over 1000 kg. Over 2000 tests per week are performed for the control testing of this material. After the first robot was installed, the release time was reduced by 50%.

This shorter release time reduces significantly the risk for Miles. A systematic problem (for example due to raw material failure) could add 30 batches a day. Reducing the time between manufacturing and product release from six days to three days reduces the number of accumulated batches with a potential problem from 180 to 90.

The first robot was set up in under three months. In the first three quarters of 1990 about 30 000 tests were run on this robot. The second is now being validated.

### The future – in-process release

Figure 1 shows the tableting machine and sealing and packaging line. The manufacturing steps shown include compression, sealing, cartoning, bundling, packaging into shippers, and transfer to the warehouse. The main test areas are hardness and weight testing, assay testing, content uniformity testing, and leakage testing to make sure the aluminium pouches are tight. Leakage would cause a product failure, because of the moisture sensitivity of the effervescent system.

The aim for the future is setting up all the testing on the lines, which means taking tablets from the tableting machine and feeding them into an automated tester. In this instrument you would test hardness and weight and give feedback information to the tableting machine; production would stop automatically if a problem was detected or even automatically correct the problem.

Also in the future it would be useful to take samples from the same tablet stream for the assay testing, content uniformity and all the other tests that are needed. Then after foiling, samples would be taken for leakage testing. Ideally, by the time the cartons are checked and the material sent to shipping, every test is done. At that point all that remains is to evaluate the data and to release the batch.

### The challenge – higher quality

The No. 1 priority in the future will be to assure even higher quality for automated systems. In quality control

© Zymark Corporation 1992

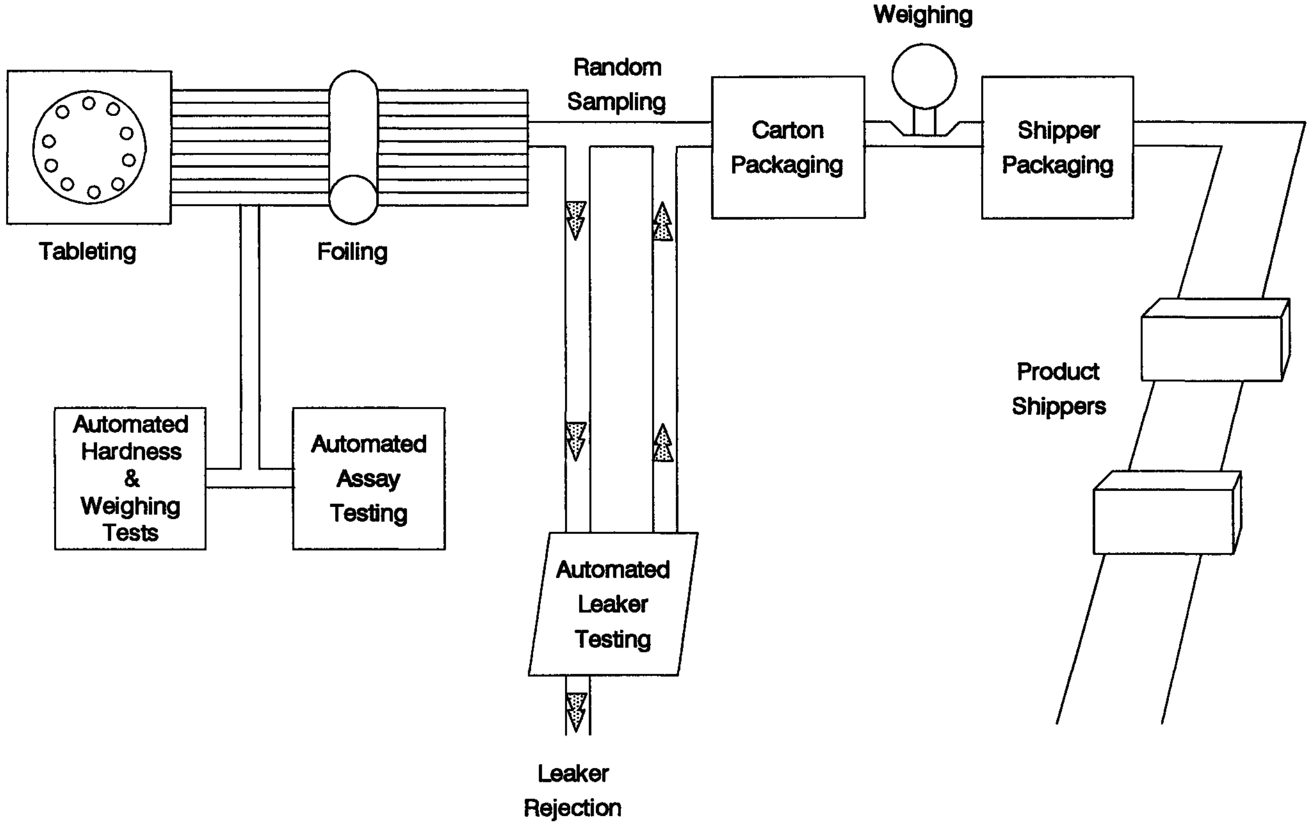

### Automated Quality Control for Effervescent Tablet Production we must be able to run this process smoothly on a daily basis. It is likely that these automated systems will, in the next few years, go through quality improvements like those of the HPLC systems in the 1970s. At the beginning of the 1980s HPLC reached a quality comparable to the UVs in the 1960s. That improvement is what is necessary here; it is an extremely high level of quality, and it is essential that everybody involved in construction, designing and building these instruments is aware of the magnitude of this requirement.

### Standardization plus flexibility

A second criterion for the use of highly automated equipment in the quality-control environment is standardization. There will be a certain level of standardization necessary so that setting up this equipment and running it will be comparable to the level of a modern HPLC. It will be essential to make it in a way that guarantees flexibility.

### Computer power will help

In addition, people will be much more creative in some other areas. For example by making more use of the already built-in intelligence in all the automated systems. It is clear that the cost for the computers which are a part of the automated system is so low that there are basically no restrictions. Today, buying sufficient memory and disk storage capacity are no longer limiting factors at all. The next challenge for our human creativity is to think about how to make optimal use of this intelligence. It is somewhat old-fashioned to install a video-camera to survey the system and subsequently evaluate what went wrong with it. The computer checks in cars are a good example of how to use the intelligent feedback. A computer hooked to the car gives the information where in former times, mechanics relied on test drives and looking at the engine, gears, etc.

### Learning from NASA

It will be necessary to go even further with new ideas and finding new ways to make this production safe and smooth. NASA or the nuclear power plants are examples of using redundant systems to increase overall reliability.

Now in the conventional quality control scenario, what happens if an implausible result is found? Usually, the test is repeated several times, because there is a good chance that the assay system had a failure in the first case. Then, repetitions can prove that the product quality is okay. If this happens again every second day in a just-in-time release system, trust in the system is lost, because it is not doing what it is expected to do.

A way to guard against this possibility is to set up redundant assay systems. One example of this approach would be the temperature sensors in nuclear power plants where they have several of them so that there is always a value with confirming data. This gives a clear indication of sensor failure or when temperature is drifting away. So putting a second automated system will immediately give the information that the first one is off and the data of the first is probably erroneous. The other one shows that the batch is okay. If a third system is added, one result or the other is quickly confirmed and just-in-time release works well. Then batch production continues uninterrupted by analytical failures.

### Gains from just-in-time release

From discussions with people at Miles it seems clear that the focus is really going significantly beyond saving labour. Of course, automation makes each test cheaper and we can be more generous with testing. It is much easier to assess to test or not to test in case of a doubt.

### In production: more flexibility, less scrap, lower risk

On top of that situation and what goes beyond it, is 'just-in-time' information. Having the just-in-time release provides the information immediately if something goes wrong at the time of manufacture. So assume that there is a content uniformity problem coming up during the run during manufacturing. It is possible to stop at that point – all the results are available to make a decision. There is at least the possibility to save packaging a poor quality batch and save all the money that was associated with that operation. Or maybe even better, that batch can be returned to production, with no loss of money in this case.

In a three day time (which is now an average release time), Miles would manufacture finished goods of about the value of $300 000. Setting up an automatic system with these advantages is something that is easily justified under the aspect of low-risk manufacturing.

### Just-in-time investigation

Together with that, I think a just-in-time investigation is another advantage that will help a lot. Since the defects are realized right at the time when the batch is being manufactured, it is possible to go back and immediately look for what went wrong. Currently, if a problem is detected, we go to the mixing area and ask Bob if there was a problem detected with the batch run on second shift last Wednesday: 'Do you remember if there was something particular about it?' It is very unlikely that Bob remembers if there was something different with that particular batch number handled three days or 90 batches ago. With just-in-time investigation, a manufacturing person can be questioned about the batch made one or two hours ago. It is much more likely that Bob may remember that when he started mixing he lost a little powder but did not think that it mattered. So the problem can be solved because it is found earlier.

To summarize my view, we are very happy because we see that our high volume production will be much safer in the future.

